# Resistance mutations of Pro197, Asp376 and Trp574 in the acetohydroxyacid synthase (AHAS) affect pigments, growths, and competitiveness of *Descurainia sophia* L

**DOI:** 10.1038/s41598-017-16655-0

**Published:** 2017-11-27

**Authors:** Yongzhi Zhang, Yufang Xu, Shipeng Wang, Xuefeng Li, Mingqi Zheng

**Affiliations:** Department of Applied Chemistry, College of Science, China Agricultural University, Beijing, 100193 China

## Abstract

*D. Sophia* is one of the most problematic weed species infesting winter wheat in China, and has evolved high resistance to tribenuron-methyl. Amino acid substitutions at site of Pro197, Asp376 and Trp574 in acetohydroxyacid synthase (AHAS) were mainly responsible for *D. sophia* resistance to tribenuron-methyl. In this study, *D. sophia* plant individually homozygous for specific AHAS mutation (Pro197Leu, Pro197His, Pro197Ser, Pro197Thr, Asp376Glu and Trp574Leu) were generated. In addition, the effects of resistance mutations on pigments, growths and competitiveness of susceptible (S) and resistant (R) plants of *D. sophia* were investigated. The results indicated the R plants carrying Pro197Leu or Pro197His or Asp376Glu or Trp574Leu displayed stronger competitiveness than S plants. The adverse effects on R plants aggravated with the increase of R plants proportion, which made the R plants against domination the weed community in absent of herbicide selection. Therefore, these resistance mutation have no obvious adverse effects on the pigments (chlorophyll a, chlorophyll b and carotenoid), relative growth rates (RGR), leaf area ratio (LAR) and net assimilation rate (NAR) of R plants.

## Introduction

Weeds are a major threat to food security, and herbicides are still the most effective tools for controlling weeds. Persistent and intensive uses exerted strong selection pressures on the weeds. In order to struggle against and survive in herbicide killings, weeds evolve resistance to herbicide, which were conferred by target-site-based (TSR) and non-target-site-based resistance (NTSR) mechanisms^1^. Therefore, weeds usually evolve resistance to herbicides at cost of improper growth or (and) reproduction, which can considerably slow the evolution of resistance and prevent the fixation of novel resistant alleles^2^.

Acetohydroxyacid synthase (AHAS; EC 2.2.1.6), also known as acetolactate synthase (ALS), is a key enzyme in the biosynthesis of branched chain amino acids (BCAAs) including valine (Val), leucine (Leu) and isoleucine (Ile). AHAS is also the target enzyme of commercial herbicides such as sulfonylurea (SU), imidazolinone (IMI), triazolopyrimidine (TP), pyrimidinylth-thiobenzoates (PTB) and sulfonylamino-carbonyl-triazolinones (SCT). AHAS-inhibiting herbicides were widely used all over the world since their first introduction in the early 1980s due to their high herbicidal activity, wide weed-control spectrum, and low mammalian toxicity. At present, 159 weed species all over the world have evolved resistance to AHAS-inhibiting herbicides owing to intensive using these herbicides^3^. In addition, the TSR mechanisms were mainly responsible for weeds resistance to AHAS-inhibiting herbicides. To date, a total of twenty-eight amino acid substitutions (numbers of amino acid in parentheses) conferring resistance to AHAS herbicides have been identified at sites (numbered according to corresponding sequence of *Arabidopsis thaliana*) of Ala122 (3), Pro197 (13), Ala205 (2), Asp 376(1), Arg377 (1), Trp574 (3), Ser653 (3) and Gly654 (2) in resistant weed biotypes^1,3–5^. Most of these resistance mutations not only altered the catalytic activity and herbicide affinity by changing 3D structure of AHAS^6–10^, but also had adverse pleiotropic effects on plant growth or (and) reproduction^11–19^.

*Descurainia sophia* L. is an annual and notorious broad-leaf weed infesting winter wheat, which has evolved extremely high resistance to SU herbicide tribenuron-methyl across China^20–22^. Our research confirmed the TSR and NTSR mechanisms conferred *D. sophia* resistance to tribenuron-methyl. Resistance mutations were identified at site of Pro197 (substituted by Ala, Leu, Thr, Ser, and His) or Asp376 (by Glu) or Trp574 (by Leu) in AHAS1 or (and) AHAS2 in tribenuron-methyl resistant *D. sophia* ^22–24^. In addition, one or more cytochrome P450s mediated *D. sophia* resistance to tribenuron-methyl^25^. Notwithstanding this, the pleiotropic effects of resistance mutations on the growths of *D. sophia* plants were not reported. The objectives of this study were to investigate the impacts of resistance mutations on: (1) the pigments contents in S and R plants; (2) classic growth of *D. Sophia* growth, such as relative growth rates (RGR), leaf area ratio (LAR), net assimilation rate (NAR) and pigment contents; (3) relative competitive ability of susceptible (S) and resistant (R) plants under condition of monoculture or admixture.

## Materials and Methods

### Plant materials

**Figure 1:**
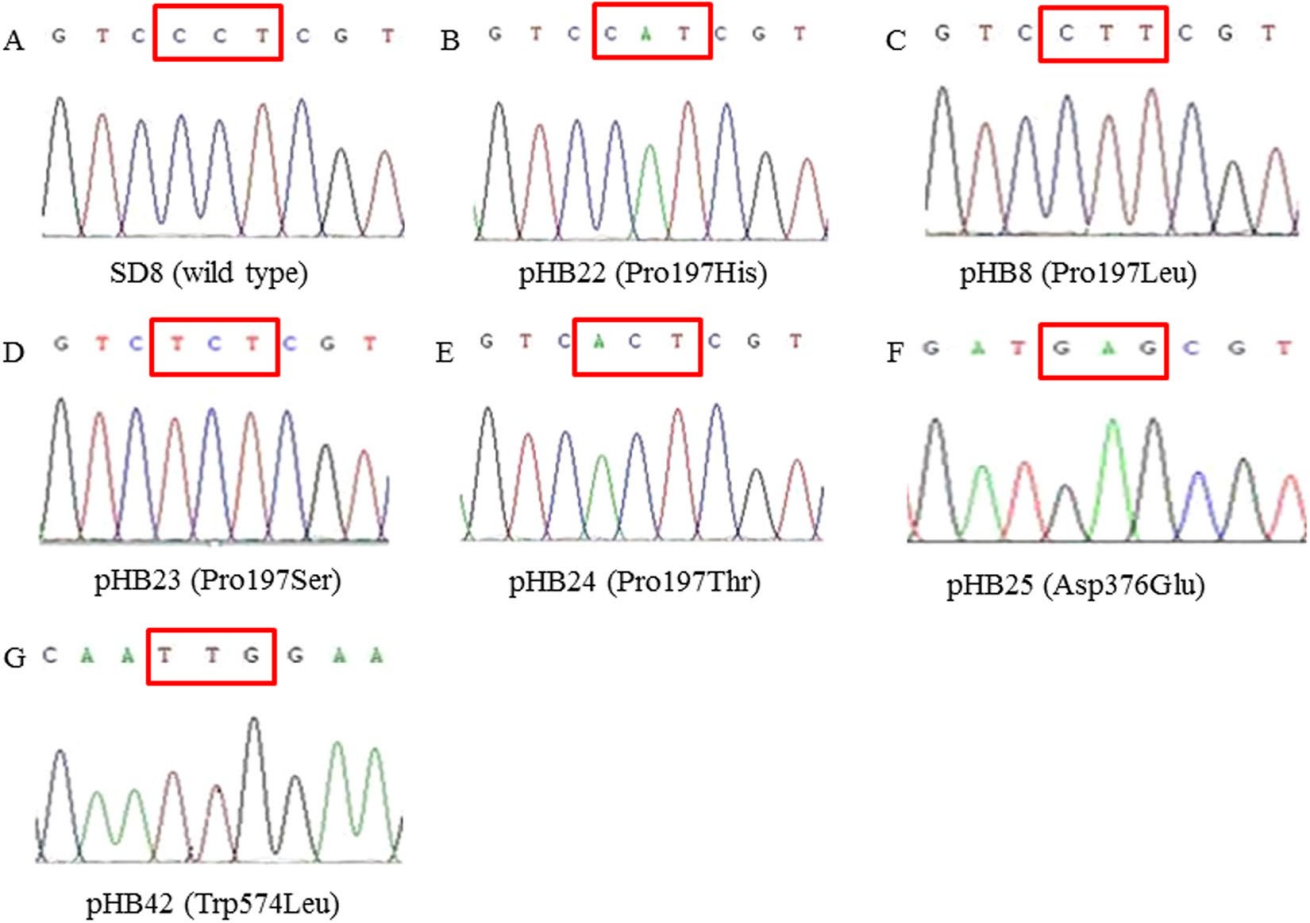
Typical sequence diagram of wild type (**A**) and purified *D. sophia* subpopulations with mutation of Pro197His (**B**), Pro197Leu (**C**), Pro197Ser (**D**), Pro197Thr (E), Asp376Glu (**F**) and Trp574Leu (**G**).

The S (SD8) *D. sophia* was collected at Linyi city of Shandong province in China that had never been treated with herbicides, which was confirmed to be individually homozygous by genotyping before. The original *D. sophia* population of each purified subpopulation was harvested from winter wheat fields in China, where tribenuron-methyl had been used continuously for at least fifteen years. In order to minimize the genetic background differences of different R plants, plants individually homozygous for specific AHAS mutation (Pro197Leu, Pro197His, Pro197Ser, Pro197Thr, Asp376Glu and Trp574Leu) were grown to generate seeds (Fig. 1). Seeds from single plants were collected separately and were pooled after genotyping. By this way, purified subpopulations individually homozygous for specific AHAS mutation (Pro197Leu, Pro197His, Pro197Ser, Pro197Thr, Asp376Glu and Trp574Leu) were obtained and used in this study (Table 1).

**Table 1:** Geographical origins of original *D. sophia* populations used for purification and generation in this study.

Purified subpopulation	Mutation in AHAS1^a^	Location	Co-ordinates	Collection years
SD8	Wild type	Linyi, Shandong Province	N35°05′45″, E118°09′03″	2013
pHB8	Pro197Leu	Handan, Hebei Province	N36°13′22″, E114°14′02″	2008
pHB22	Pro197His	Baoding, Hebei Province	N38°55′48″, E115°33′19″	2014
pHB23	Pro197Ser	Baoding, Hebei Province	N38°48′55″, E115°33′23″	2014
pHB24	Pro197Thr	Dingzhou, Hebei Province	N38°45′2.8″, E115°28′40″	2014
pHB25	Asp376Glu	Baoding, Hebei Province	N38°39′58″, E115°26′34″	2014
pHB42	Trp574Leu	Dingzhou, Hebei Province	N38°35′04″, E115°01′35″	2014

^a^AHAS2 isozyme is wild type and found in all *D. sophia* population.

Seeds of purified S and R subpopulations were immersed in 30% hydrogen peroxide solution for 35 min, and then soaked in 0.03% gibberellins solution for 24 h after rinsing with distilled water. Then, seeds were placed in Petri dishes to germinate for 96 h. Germinating seedlings with similar size were selected and transplanted into square plastic pots (7.5 cm slides) containing moist loam soil, and were brought up in an artificial climate chamber under conditions of 25 °C/15 °C (light/dark), 14 h photoperiod with luminous intensity of 15,000 lx. *D. sophia* plants were watered and rearranged regularly to minimize the environmental effects on the plants growth.

### Whole-plant response experiments to tribenuron-methyl

In order to confirm the resistance to tribenuron-methyl of each purified R subpopulation, seedlings at 14 days after transplant (DAT) were used for whole-plant response experiment. Plants were returned to artificial climate chamber after herbicide treatment, and the above-ground shoots were harvested 21 days later. The above-ground shoots were oven dried for 96 h at 65 °C. The experiment was conducted with three replicates per herbicide dose and repeated twice.

Tribenuron-methyl was applied to S (5.7 × 10^−4^, 2.3 × 10^−3^, 9.2 × 10^−3^, 3.7 × 10^−2^, 0.15, 0.59, 2.3, 9.4 g a. i. ha^−1^) and R (0.15, 0.59, 2.3, 9.4, 37.5, 75, 150 g a. i. ha^−1^) subpopulations using a moving-boom cabinet sprayer delivering 600 L ha^−1^ water at a pressure of 0.4 MPa by a flat fan nozzle positioned 54 cm above the foliage.

### Determination of the pigment content (chlorophyll a, chlorophyll b and carotenoid) in S and R plants

The extraction and determination of chlorophyll a, chlorophyll b and carotenoid were conducted according the methods described by Wellburn (1994)^26^. Above-ground seedling of individual plant at 35 or 50 DAT was ground to fine powder with a mortar and pestle in liquid nitrogen, and then homogenized in 3 mL water solutions containing 80% acetone. Six plants of S or each R subpopulation were used for pigment test. The homogenate was filtered into a brown bottle through filter paper wetting with 80% acetone. The chloroplast pigment on the filter paper was washed with 80% acetone solutions, and combined with the filtrate. The filtrate was adjusted to 25 mL by adding 80% acetone, and used for following test. Six plants were selected from each subpopulation and used for pigment extraction.

The absorbance value of each filtrate at 470, 646 and 663 nm was determined with Lambda 35 spectrophotometer (Perkins-Elmer). The concentration of chlorophyll a, chlorophyll b and carotenoid was calculated respectively by formulas (1), (2) and (3). The total chlorophyll is the sums chlorophyll a and b.

1 $${\rm{Ca}}=12{{\rm{.21A}}}_{663}-2{{\rm{.81A}}}_{646}$$

2 $${\rm{Cb}}=20.13{{\rm{A}}}_{646}-5.03{{\rm{A}}}_{663}$$

3 $${\rm{Cc}}=(1{{\rm{000A}}}_{470}-3.{\rm{27Ca}}-{\rm{104Cb}})/229$$

where Ca, Cb and Cc are the concentration of chlorophyll a, chlorophyll b and carotenoids respectively; A_663_, A_646_ and A_470_ are the absorbance value at wavelengths of 663, 646 and 470 nm respectively.

The contents of various pigments in a unit of fresh weight of the tissue were calculated by following formula.

$${\rm{A}}={\rm{n}}\cdot {\rm{C}}\cdot {\rm{N}}\cdot {\rm{W}}$$

where A is the content of a pigments; C is the concentration of pigments; n- is the volume of extraction solution; N is the dilution ratio; W is the fresh weight of sample.

### Determination of RGR, LAR and NAR of S and R plants

The above-ground shoots were harvested respectively at 23, 29, 34, 39, 45, 55 and 63-day stage. The above-ground shoots were oven dried for 96 h at 65 °C, and dry weight was measured. The leaf area of each plant was measured immediately after harvest.

The unbiased RGR was estimated by the formula proposed by Hoffmann and Poorter (2002)^27^. RGR = (ln ${\overline{{\rm{W}}}}_{2}$ − ln ${\overline{{\rm{W}}}}_{1}$)/(t_2_ − t_1_). Where ${\overline{{\rm{W}}}}_{1}$ and ${\overline{{\rm{W}}}}_{2}$ are means of dry weight per plant at times t_1_ and t_2_. The ln ${\overline{{\rm{W}}}}_{1}$ and ln ${\overline{{\rm{W}}}}_{2}$ are the natural logarithm-transformed means of dry weight per plant.

Leaf area per plant was measured with Photoshop CS3 extended (Adobe Systems Inc., USA). LAR was calculated by the formula proposed by Hunt (1982)^28^. LAR = [(ln ${\overline{{\rm{W}}}}_{2}$ − ln ${\overline{{\rm{W}}}}_{1}$)(${\bar{{\rm{L}}}}_{{\rm{A2}}}$ − ${\bar{{\rm{L}}}}_{{\rm{A1}}}$)]/[(${\overline{{\rm{W}}}}_{2}$ − ${\overline{{\rm{W}}}}_{1}$)(ln ${\bar{{\rm{L}}}}_{{\rm{A2}}}$ − ln ${\bar{{\rm{L}}}}_{{\rm{A1}}}$)]. Where ${\overline{{\rm{W}}}}_{1}$ and ${\overline{{\rm{W}}}}_{2}$ are means of dry weight per plant at times t_1_ and t_2_, ${\bar{{\rm{L}}}}_{{\rm{A1}}}$ and ${\bar{{\rm{L}}}}_{{\rm{A2}}}$ are means of leaf area per plant at t_1_ and t_2_. The ln ${\overline{{\rm{W}}}}_{1}$ and ln ${\overline{{\rm{W}}}}_{2}$ are the natural logarithm-transformed means of dry weight per plant, ln ${\bar{{\rm{L}}}}_{{\rm{A2}}}$ and ln ${\bar{{\rm{L}}}}_{{\rm{A2}}}$ are the natural logarithm-transformed means of leaf area per plant.

NAR was estimated by the formula proposed by Hunt (1982). NAR = [(${\bar{{\rm{W}}}}_{2}$ − ${\bar{{\rm{W}}}}_{1}$) (ln $\bar{{\rm{W}}}\,$_2_ − ln $\bar{{\rm{W}}}{}_{1}$)]/[(${\bar{{\rm{L}}}}_{{\rm{A2}}}$ − ${\bar{{\rm{L}}}}_{{\rm{A1}}}$) (t_2_−t_1_)]^28^. Where $\bar{{\rm{W}}}{}_{1}$ and ${\bar{{\rm{W}}}}_{2}$ are means of dry weight per plant at times t_1_ and t_2_, ${\bar{{\rm{L}}}}_{{\rm{A1}}}$ and ${\bar{{\rm{L}}}}_{{\rm{A2}}}$ are means of leaf area per plant at t_1_ and t_2_. The ln $\bar{{\rm{W}}}{}_{1}$ and ln ${\bar{{\rm{W}}}}_{2}$ are the natural logarithm-transformed means of dry weight per plant.

### Relative competition ability of S and R plants under condition of monoculture

Individual seedling was transplanted into a square plastic pot (7.5 cm slides), and harvested at 35 and 50 DAT respectively. The leaf area of each plant was measured immediately after collection. The above-ground shoots were oven dried for 96 h at 65 °C, and dry weight was measured. Total 20 plants from each S or R subpopulation were selected for test.

### Relative competition ability of S and R plants under condition of admixture

Relative competition ability between S and R plants was evaluated using a replacement series experiment (S:R = 100:0, 75:25, 50:50, 25:75, 0:100) at a constant density of 644 plants m^−2^ (24 plants per tray, 23.3 cm × 16.0 cm × 6.0 cm) according the methods described by Reboud *et al*.^29^. The experiment was conducted in a randomized complete block design with four replications. The above-ground shoots of S or R plants in the same tray were harvested separately at 50 DAT. The leaf area of each plant was measured immediately after harvest. The above-ground shoots were oven dried for 96 h at 65 °C, and dry weight of above-ground biomass was measured.

The relative crowding coefficient (RCC) was calculated according to the following formula^17,30^.

$$\begin{array}{c}{\rm{RCC}}=(\{[{{{\rm{DB}}}_{{\rm{S}}}}^{75:25}/{{{\rm{DB}}}_{{\rm{R}}}}^{75:25}]+({{{\rm{DB}}}_{{\rm{S}}}}^{50:50}/{{{\rm{DB}}}_{{\rm{R}}}}^{50:50})+[{{{\rm{DB}}}_{{\rm{S}}}}^{25:75}/{{{\rm{DB}}}_{{\rm{R}}}}^{25:75}]/N\})\\ \,\,\,\,\,\,\,\,\,\,\,\,\,\,\,(D{{B}_{S}}^{100:0}/D{{B}_{R}}^{0:100})\end{array}$$

where, DB_S_^n:n^ and DB_R_^n:n^ is the mean dry biomass per S and R plant respectively at ratio of n:n, N is the number of mixed plantings; here N = 3. Based on this definition, an RCC value greater than 1.0 indicate S is superior competitiveness over R; an RCC value lower than 1.0 shows R is outcompeting S; While, an RCC value around 1.0 indicates S and R have similar competition ability.

### Statistical analyses

The data of whole-plant response experiments was converted into the percentage of control and subjected to a non-linear regression analysis using GraphPad Software (v.5.0)^31,32^. In this study, two AHAS isozymes with different sensitivities to tribenuron-methyl were confirmed to coexist in all R (pHB8, pHB22, pHB23, pHB24, pHB25 and pHB42) subpopulations. The GR_50_ (herbicide concentrations causing 50% plant growth reduction) values for all S and R subpopulations were calculated using single-sigmoid equation *f(x)*.

1 $$y=p\times f(x)+(1-p)\times g(x)$$

$$f(x)=100/(1+{10}^{(a-x)})$$

$$g(x)=100/(1+{10}^{(b-x)})$$

One-way analysis of variance (ANOVA) with Dunnett’s post-test (α = 5%) was performed to assess pairwise differences in plant growth (RGR, NAR, LAR, BCAAs content and relative competition ability).

### Data Availability

The datasets generated during and/or analysed during the current study are available from the corresponding author on reasonable request.

## Results

### Whole-plant response experiments to confirm resistance to tribenuron-methyl in each R subpopulation

**Figure 2:**
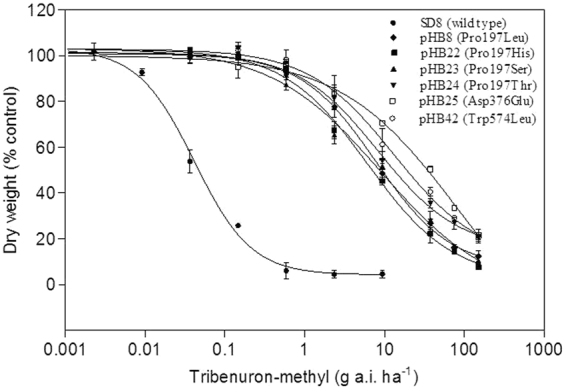
Dose-response curves to tribenuron-methyl for the S and R *D. sophia* subpopulations with mutation of Pro197Leu, Pro197His, Pro197Ser, Pro197Thr, Asp376Glu and Trp574Leu. Each point is the means ± SE of two experiments, each experiment containing three replicates.

The whole-plant response experiments established that all R subpopulations exhibited extremely high resistance levels to tribenuron-methyl (Fig. 2). The pHB25 subpopulation carrying Asp376Glu mutation exhibited the highest resistance level to tribenuron-methyl, with the resistance index (RI) of 815. The RI value of pHB42 subpopulation (with Trp574Leu) was 366.3, which was higher than all plants carrying Pro197 mutations (Pro197Leu, His, Thr and Ser). In contrast, the resistance level of R subpopulations carrying different Pro197 mutations displayed no significant differences.

### Effects of resistance mutations on the pigments (chlorophyll a, chlorophyll b and carotenoid) in S and R plants

At stage of 35 DAT, only Pro197His (pHB22) significantly reduce the pigment contents (carotenoid, chlorophyll a, chlorophyll b and total chlorophyll) in *D. sophia* plants, while the other resistance mutations have no obvious effects on corresponding R plants. In contrast to results of 35 DAT, the pigment contents in S and all R subpopulations at stage of 50 DAT exhibited no significant differences (Table 2).

**Table 2:** Pigments content (chlorophyll a, b and carotenoid) of S (SD8) and R *D. sophia* subpopulations carrying different resistance-endowing mutations at 35 and 50 DAT.

Purified subpopulation	Mutation in AHAS1	chlorophyll a (mg·g^−1^)		chlorophyll b (mg·g^−1^)		Total chlorophyll (mg·g^−1^)		Carotenoids (mg·g^−1^)	
		35 DAT^a^	50 DAT	35 DAT	50 DAT	35 DAT	50 DAT	35 DAT	50 DAT
SD8	Wild type	1.19 ± 0.065^a^	1.57 ± 0.26^a^	0.42 ± 0.045^ab^	0.48 ± 0.0060^a^	1.61 ± 0.099^a^	2.05 ± 0.029^a^	0.256 ± 0.016^a^	0.35 ± 0.007^a^
pHB8	Pro197Leu	1.25 ± 0.018^a^	1. ± 0.040^a^	0.39 ± 0.007^ab^	0.45 ± 0.024^a^	1.64 ± 0.022^a^	1.80 ± 0.064^a^	0.288 ± 0.007^a^	0.32 ± 0.010^a^
pHB22	Pro197His	1.57 ± 0.222^b^	1.48 ± 0.054^a^	0.55 ± 0.055^b^	0.47 ± 0.0090^a^	2.07 ± 0.279^b^	1.95 ± 0.064^a^	0.363 ± 0.054^b^	0.34 ± 0.017^a^
pHB23	Pro197Ser	1.26 ± 0.036^a^	1.64 ± 0.036^a^	0.37 ± 0.011^a^	0.49 ± 0.0090^a^	1.64 ± 0.046^a^	2.13 ± 0.043^a^	0.300 ± 0.010^a^	0.38 ± 0.007^a^
pHB24	Pro197Thr	1.34 ± 0.124^a^	1.64 ± 0.315^a^	0.42 ± 0.050^ab^	0.48 ± 0.010^a^	1.52 ± 0.264^a^	2.12 ± 0.424^a^	0.305 ± 0.020^a^	0.37 ± 0.079^a^
pHB25	Asp376Glu	1.17 ± 0.050^a^	1.41 ± 0.057^a^	0.40 ± 0.041^ab^	0.42 ± 0.027^a^	1.56 ± 0.089^a^	1.83 ± 0.082^a^	0.253 ± 0.008^a^	0.33 ± 0.020^a^
pHB42	Trp574Leu	1.25 ± 0.015^a^	1.36 ± 0.038^a^	0.40 ± 0.019^ab^	0.40 ± 0.027^a^	1.65 ± 0.009^a^	1.76 ± 0.065^a^	0.286 ± 0.011^a^	0.31 ± 0.010^a^

Within a column, pigment values with different letters are significantly different at P = 0.05 significant levels. Data were means ± SE of four replicates. ^a^DAT, days after transplant.

### Effects of resistance mutations on RGR, LAR and NAR of S and R plants

The Pro197Leu and Pro197Thr reduced plant growth during all tested periods, and decreased RGR about 13% and 23% during period of 23–63 DAT respectively. The other mutations decreased RGR only at partial stages. In addition, all mutations had adverse pleiotropic effects on plant growth during periods of 23–34 and 23–39 DAT. While, only partial mutations had negative impacts on RGR during other periods (Table 3).

**Table 3:** Relative growth rates (RGR) of S (SD8) and R *D. sophia* subpopulations carrying different resistance-endowing mutations at different growth periods.

Purified subpopulation	Mutation in AHAS1	RGR (mg mg^−1^ day^−1^)					
		23–29 DAT^a^	23–34 DAT	23–39 DAT	23–45 DAT	23–55 DAT	23–63 DAT
SD8	Wild type	0.16 ± 0.023^a^	0.18 ± 0.0073^a^	0.14 ± 0.0062^a^	0.10 ± 0.003^a^	0.095 ± 0.0018^ab^	0.098 ± 0.0034^a^
pHB8	Pro197Leu	0.075 ± 0.026^bc^	0.057 ± 0.018 e	0.056 ± 0.012^d^	0.057 ± 0.0044^d^	0.076 ± 0.0055^c^	0.085 ± 0.00091^b^
pHB22	Pro197His	0.15 ± 0.024^a^	0.13 ± 0.0037^bc^	0.10 ± 0.0056^b^	0.082 ± 0.0073^c^	0.098 ± 0.0031^ab^	0.092 ± 0.0049^ab^
pHB23	Pro197Ser	0.022 ± 0.0045^c^	0.12 ± 0.014^bcd^	0.095 ± 0.009^bc^	0.09 ± 0.003 bc	0.095 ± 0.0056^ab^	0.088 ± 0.004^ab^
pHB24	Pro197Thr	0.042 ± 0.015^bc^	0.092 ± 0.0073^d^	0.068 ± 0.0095^cd^	0.067 ± 0.0055^d^	0.070 ± 0.0036^c^	0.075 ± 0.0028^c^
pHB25	Asp376Glu	0.088 ± 0.021^abc^	0.14 ± 0.012^b^	0.10 ± 0.0038^b^	0.10 ± 0.0026^ab^	0.089 ± 0.004^b^	0.090 ± 0.0034^ab^
pHB42	Trp574Leu	0.10 ± 0.031^ab^	0.11 ± 0.008^cd^	0.084 ± 0.007^bc^	0.11 ± 0.0042^a^	0.10 ± 0.0024^a^	0.096 ± 0.0027^ab^

Within ^a^column, RGR values with different letters are significantly different at P = 0.05 significant levels. Data were means ± SE of four replicates. ^a^DAT, days after transplant.

For the LAR and NAR during 23–63 DAT, only Trp574Leu increased the LAR (about 29%), while the other mutations had no obvious pleiotropic effects on LAR and NAR. In addition, the Trp574Leu increased the LAR during all tested periods, all mutations decreased the NAR during period of 23–39 DAT (Tables 4 and 5).

**Table 4:** Leaf area ratios (LAR) of S (SD8) and R *D. sophia* subpopulations carrying different resistance-endowing mutations at different growth periods.

Purified subpopulation	Mutation in AHAS1	LAR (cm^2^ mg^−1^)					
		23–29 DAT^a^	23–34 DAT	23–39 DAT	23–45 DAT	23–55 DAT	23–63 DAT
SD8	Wild type	0.53 ± 0.0074^a^	0.32 ± 0.043^a^	0.51 ± 0.011^a^	0.52 ± 0.024^a^	0.44 ± 0.016^abc^	0.42 ± 0.027^ab^
pHB8	Pro197Leu	0.66 ± 0.043^b^	0.59 ± 0.068^c^	0.56 ± 0.049^ab^	0.55 ± 0.010^ab^	0.52 ± 0.0061^c^	0.46 ± 0.017 abc
pHB22	Pro197His	0.58 ± 0.006^ab^	0.42 ± 0.022^ab^	0.59 ± 0.016^ab^	0.54 ± 0.044^ab^	0.43 ± 0.045^ab^	0.41 ± 0.032^a^
pHB23	Pro197Ser	0.62 ± 0.025^ab^	0.35 ± 0.053^ab^	0.51 ± 0.026^a^	0.53 ± 0.032^ab^	0.40 ± 0.019^a^	0.46 ± 0.027^abc^
pHB24	Pro197Thr	0.61 ± 0.079^ab^	0.42 ± 0.025^ab^	0.63 ± 0.026^bc^	0.61 ± 0.020^bc^	0.53 ± 0.044^d^	0.51 ± 0.019^bc^
pHB25	Asp376Glu	0.62 ± 0.017^ab^	0.34 ± 0.022^ab^	0.60 ± 0.021^b^	0.55 ± 0.017^ab^	0.49 ± 0.014^bcd^	0.52 ± 0.048^bc^
pHB42	Trp574Leu	0.65 ± 0.025^b^	0.46 ± 0.022^b^	0.70 ± 0.025^c^	0.64 ± 0.018^c^	0.54 ± 0.010^d^	0.54 ± 0.023^c^

Within a column, LAR values with different letters are significantly different at P = 0.05 significant levels. Data were means ± SE of four replicates. ^a^DAT, days after transplant.

**Table 5:** Net assimilation rate (NAR) of S (SD8) and R *D. sophia* subpopulations carrying different resistance-endowing mutations at different growth periods.

Purified subpopulation	Mutation in AHAS1	NAR (mg cm^−2^ d^−1^)					
		23–29 DAT^a^	23–34 DAT	23–39 DAT	23–45 DAT	23–55 DAT	23–63 DAT
SD8	Wild type	0.25 ± 0.049^ab^	0.79 ± 0.13^ab^	0.28 ± 0.023^a^	0.20 ± 0.019^a^	0.24 ± 0.013^ab^	0.26 ± 0.034^a^
pHB8	Pro197Leu	0.088 ± 0.008^c^	0.23 ± 0.11^b^	0.05 ± 0.0067^c^	0.10 ± 0.005^b^	0.15 ± 0.011^b^	0.20 ± 0.012^a^
pHB22	Pro197His	0.26 ± 0.039^ab^	0.59 ± 0.12^b^	0.17 ± 0.014^b^	0.20 ± 0.029^a^	0.28 ± 0.052^a^	0.27 ± 0.041^a^
pHB23	Pro197Ser	0.073 ± 0.030^c^	0.48 ± 0.21^b^	0.19 ± 0.036^b^	0.18 ± 0.026^a^	0.28 ± 0.033^a^	0.26 ± 0.071^a^
pHB24	Pro197Thr	0.068 ± 0.031^c^	0.66 ± 0.16^ab^	0.12 ± 0.031 bc	0.12 ± 0.011^b^	0.16 ± 0.034^b^	0.17 ± 0.015^a^
pHB25	Asp376Glu	0.15 ± 0.044^bc^	1.20 ± 0.51^a^	0.17 ± 0.015^b^	0.20 ± 0.011^a^	0.21 ± 0.017 ab	0.19 ± 0.027^a^
pHB42	Trp574Leu	0.30 ± 0.058^a^	0.50 ± 0.069^b^	0.12 ± 0.017^bc^	0.18 ± 0.015^a^	0.21 ± 0.006^ab^	0.20 ± 0.016^a^

Within a column, NAR values with different letters are significantly different at P = 0.05 significant levels. Data were means ± SE of four replicates. ^a^DAT, days after transplant.

### Relative competition ability of S and R plants under monoculture (one plant per pot)

At stage of 50 DAT, the dry weight of S plants increased about 56%, 46%, 42%, 18% and 70% than R plants carrying mutation of Pro197Leu, Pro197His, Pro197Thr, Asp376Glu and Trp574Leu, respectively. While the S and R plants (with Pro197Ser) displayed no significant differences in terms of dry weight. By contrast to plants at 50 DAT, the effects of resistance mutation on S and R plants at 35 DAT were complex. For example, the Pro197His and Trp574Leu significantly decreased the dry weight of R plants; Pro197Leu, Pro197Thr and Asp376Glu did not change the dry weight of R plants comparing with S plants. Therefore, the Pro197Ser mutation significantly increased the dry weight of R plants (Table 6).

**Table 6:** Dry weight and leaf area of S (SD8) or R *D. sophia* plant carrying different resistance mutations at 35 and 50 DAT under monoculture condition.

Purified subpopulation	Mutation in AHAS1	Dry weight (g per plant)		Leaf area (cm^2^ per plant)	
		35 DAT^a^	50 DAT	35 DAT	50 DAT
SD8	Wild type	3.36 ± 0.46^bc^	23.39 ± 1.20^a^	1.63 ± 0.11^bc^	8.94 ± 0.37^a^
pHB8	Pro197Leu	3.92 ± 0.18^c^	10.36 ± 0.66^d^	2.13 ± 0.081^c^	5.43 ± 0.27^c^
pHB22	Pro197His	1.52 ± 0.12^a^	12.66 ± 1.12^cd^	0.89 ± 0.057^a^	6.62 ± 0.49^b^
pHB23	Pro197Ser	5.06 ± 0.26^d^	22.04 ± 1.50^ab^	2.58 ± 0.15^e^	8.10 ± 0.36^a^
pHB24	Pro197Thr	2.99 ± 0.19^b^	13.60 ± 1.02^c^	1.44 ± 0.096^b^	5.20 ± 0.28^c^
pHB25	Asp376Glu	2.98 ± 0.11^b^	19.18 ± 0.98^b^	1.88 ± 0.058^cd^	8.20 ± 0.39^a^
pHB42	Trp574Leu	1.34 ± 0.11^a^	6.96 ± 0.66^e^	0.75 ± 0.044^a^	3.81 ± 0.31^d^

Within a row, individual above-ground dry weights values with different letters are significantly different at P = 0.05 significant levels. Data were means ± SE of four replicates. ^a^DAT, days after transplant.

In contrast to dry weights, the individual leaf area of S at 50 DAT was about 39%, 26%, 42% and 57% larger than R plants carrying Pro197Leu, Pro197His, Pro197Thr and Trp574Leu, respectively. In addition, the individual leaf area of S (35 DAT) was about 45% and 53% larger than R plants with Pro197His and Trp574Leu, respectively. Therefore, the individual leaf area of S (35 DAT) displayed no significant differences with R plants carrying Pro197Leu, Pro197Thr and Asp376Glu (Table 6).

### Relative competition ability of S and R plants under admixture condition

**Figure 3:**
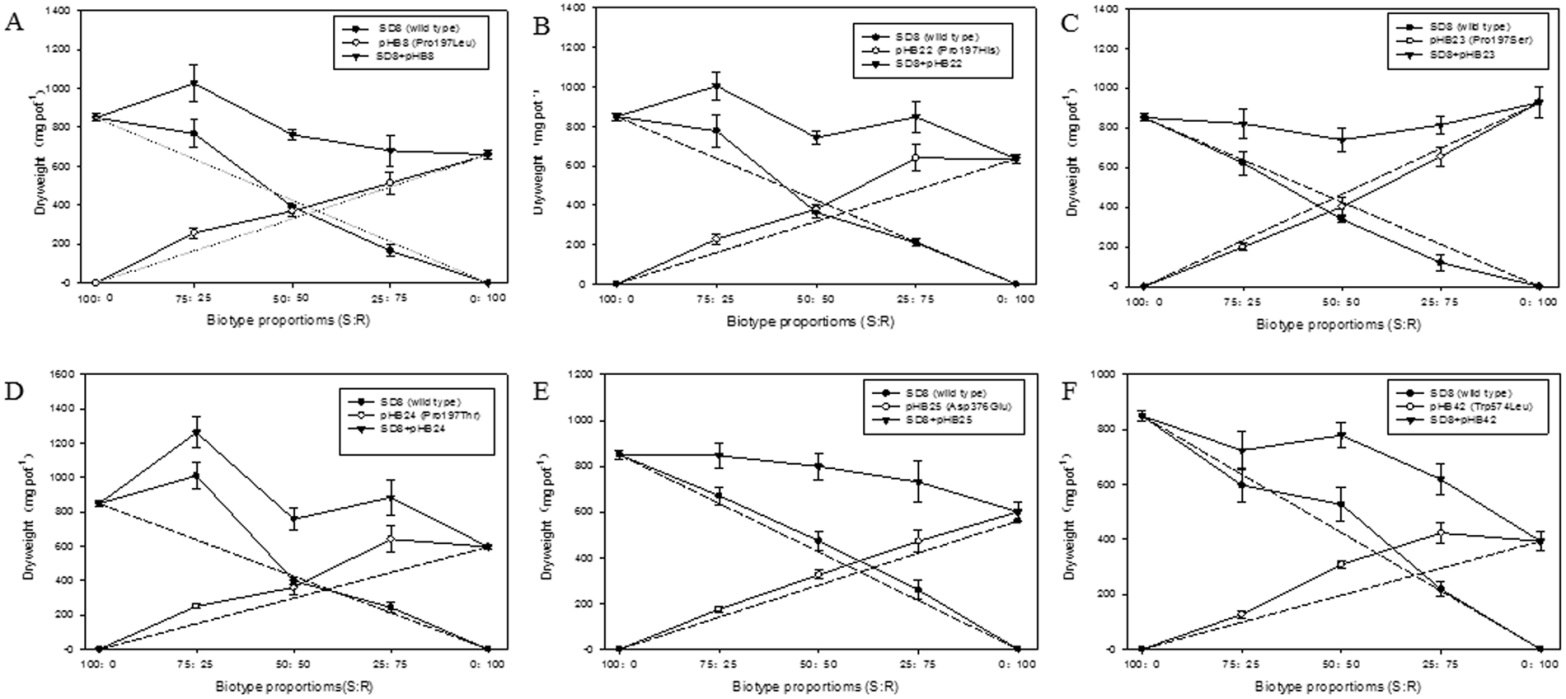
The individual and both dry weights of S or (and) R *D. sophia* plants carrying mutation of Pro197Leu (**A**), Pro197His (**B**), Pro197Ser (**C**), Pro197Thr (**D**), Asp376Glu (**E**) and Trp574Leu (**F**). Plants of S and R biotypes were grown at proportion of 100:0, 75:25, 50:50, 25:75 and 0:100. Each point is the means ± SE of four replicates.

**Figure 4:**
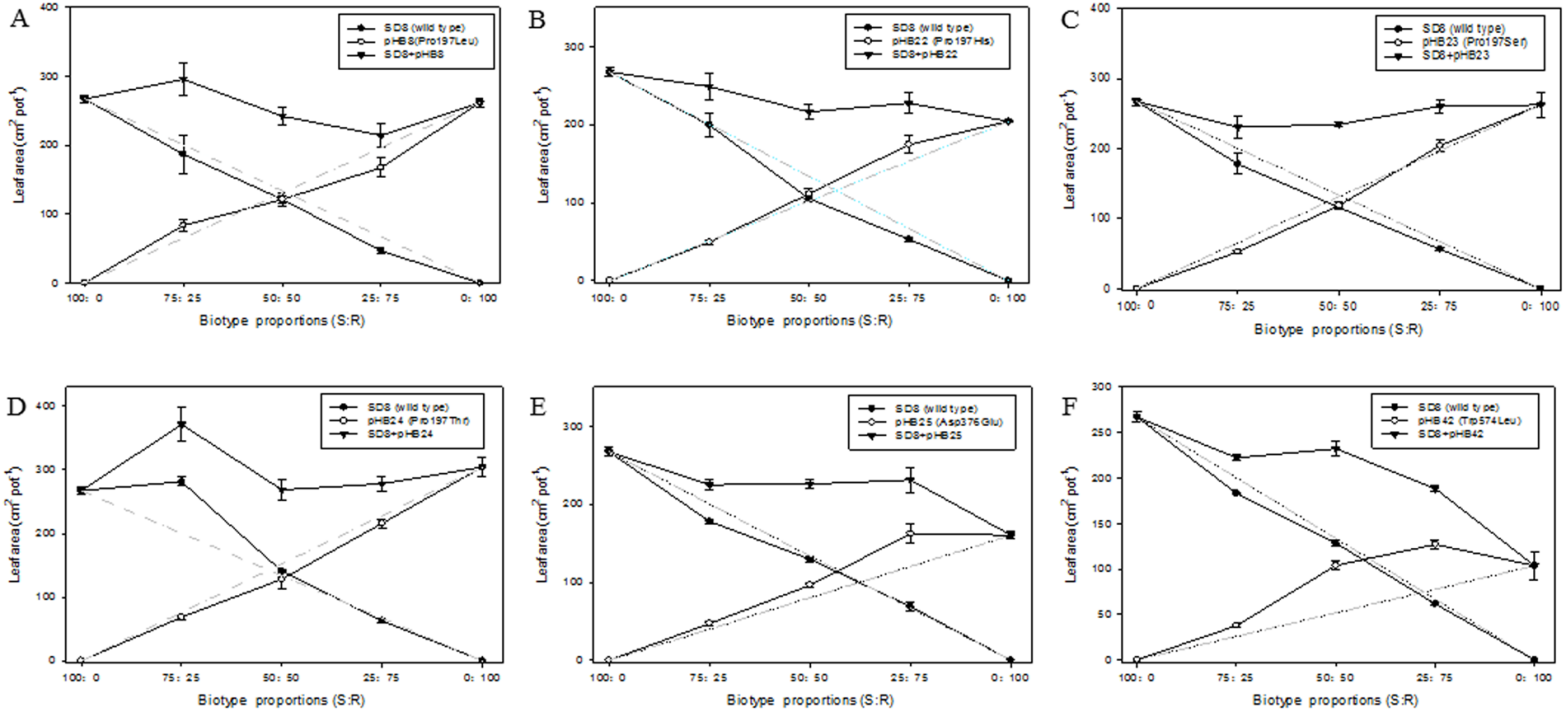
The individual and both leaf areas of S or (and) R *D. sophia* plants carrying mutation of Pro197Leu (**A**), Pro197His (**B**), Pro197Ser (**C**), Pro197Thr (**D**), Asp376Glu (**E**) and Trp574Leu (**F**). Plants of S and R biotypes were grown at proportion of 100:0, 75:25, 50:50, 25:75 and 0:100. Each point is the means ± SE of four replicates.

When mixed with R plants (with Pro197Leu or Pro197His or Pro197Thr or Asp376Glu or Trp574Leu), the dry weight of S unchanged significantly at all proportions. While the dry weight of R plants reduced greatly with increasing of R proportion to 75%~100%. By contrast, dry weight of S plants decreased at proportion of 25:75 (S:R), while pHB23 plants (with Pro197Ser) unchanged at all proportions (Tables 7 and 8 and Figs 3 and 4).

**Table 7:** Individual above-ground dry weights of S (SD8) and R *D. sophia* plant carrying different resistance-endowing mutations in replacement series experiment at proportions of 100:0, 75:25, 50:50, 25:75 and 0:100 (S:R).

Groups	Purified subpopulation	Biotype	Mutation in AHAS1	Dry weight of S and R biotypes (mg per plant)(S:R)					RCC^a^
				100:0	75:25	50:50	25:75	0:100	
1	SD8	S	Wild type	35.41 ± 0.78^ab^	36.43 ± 2.33^ab^	32.76 ± 0.75^ab^	27.71 ± 5.02^a^	0	0.77
	pHB8	R	Pro197Leu	0	42.86 ± 4.45^a^	30.78 ± 2.22^b^	28.55 ± 3.04^b^	27.49 ± 1.06^b^	
2	SD8	S	Wild type	35.41 ± 0.78^a^	36.14 ± 1.96^a^	30.22 ± 2.05^a^	34.76 ± 3.08^a^	0	0.72
	pHB22	R	Pro197His	0	33.85 ± 1.97^a^	31.66 ± 1.83^a^	32.30 ± 2.68^a^	24.70 ± 1.90^b^	
3	SD8	S	Wild type	35.41 ± 0.78^ab^	31.82 ± 1.02^ab^	28.17 ± 1.36^bc^	26.65 ± 2.45^c^	0	0.92
	pHB23	R	Pro197Ser	0	33.06 ± 2.88^a^	33.39 ± 3.70^a^	36.31 ± 2.55^a^	38.64 ± 3.13^a^	
4	SD8	S	Wild type	35.41 ± 0.78^a^	50.97 ± 3.41^a^	33.22 ± 1.74^a^	33.36 ± 11.32^a^	0	0.62
	pHB24	R	Pro197Thr	0	47.55 ± 5.99^a^	29.99 ± 3.49^b^	25.60 ± 4.31^b^	21.17 ± 2.68^b^	
5	SD8	S	Wild type	35.41 ± 0.78^a^	37.28 ± 2.16^a^	39.44 ± 3.60^a^	37.84 ± 5.75^a^	0	0.86
	pHB25	R	Asp376Glu	0	26.71 ± 0.68^a^	28.62 ± 0.64^a^	28.52 ± 2.21^a^	22.29 ± 1.11^b^	
6	SD8	S	Wild type	35.41 ± 0.78^a^	33.19 ± 3.41^a^	44.02 ± 5.12^a^	36.46 ± 4.44^a^	0	0.73
	pHB42	R	Trp574Leu	0	23.84 ± 3.02^a^	25.72 ± 1.29^a^	22.20 ± 1.93^ab^	16.37 ± 1.43^b^	

In a row, individual above-ground dry weights values with different letters are significantly different at P = 0.05 significant levels. Data were means ± SE of four replicates. ^a^RCC refers to relative crowding coefficient. ^a^RCC refers to relative crowding coefficient.

**Table 8:** Individual leaf area of S (SD8) and R *D. sophia* plant carrying different resistance-endowing mutations in replacement series experiment at proportions of 100:0, 75:25, 50:50, 25:75 and 0:100 (S:R).

Groups	Purified subpopulation	Biotype	Mutation in AHAS1	Leaf area of S and R biotypes (cm^2^ per plant)(S:R)					RCC^a^
				100:0	75:25	50:50	25:75	0:100	
1	SD8	S	Wild type	11.14 ± 0.23^a^	10.66 ± 0.44^a^	10.10 ± 0.40^a^	7.82 ± 0.75^b^	0	0.77
	pHB8	R	Pro197Leu	0	13.92 ± 1.52^b^	10.09 ± 0.82^a^	10.08 ± 0.67^a^	10.16 ± 0.77^a^	
2	SD8	S	Wild type	11.14 ± 0.23^a^	10.25 ± 0.54^a^	8.74 ± 0.31^b^	8.85 ± 0.49^b^	0	0.74
	pHB22	R	Pro197His	0	8.25 ± 0.51^a^	9.29 ± 0.56^a^	9.69 ± 0.63^a^	7.97 ± 0.53^a^	
3	SD8	S	Wild type	11.14 ± 0.23^a^	9.89 ± 0.15^b^	9.66 ± 0.04^b^	9.35 ± 0.36^b^	0	0.96
	pHB23	R	Pro197Ser	0	8.76 ± 0.30^b^	9.89 ± 0.38^ab^	11.35 ± 0.43^a^	10.94 ± 0.73^a^	
4	SD8	S	Wild type	11.14 ± 0.23^a^	15.43 ± 0.34^b^	11.70 ± 0.08^a^	10.48 ± 0.62^a^	0	1.28
	pHB24	R	Pro197Thr	0	11.42 ± 0.62^a^	10.23 ± 1.03^a^	11.99 ± 0.37^a^	12.68 ± 1.87^a^	
5	SD8	S	Wild type	11.14 ± 0.23^a^	10.28 ± 0.11^a^	10.79 ± 0.27^a^	11.47 ± 0.93^a^	0	0.76
	pHB25	R	Asp376Glu	0	7.89 ± 0.53^b^	8.02 ± 0.21^b^	9.01 ± 0.67^b^	6.46 ± 0.26^a^	
6	SD8	S	Wild type	11.14 ± 0.23^a^	10.83 ± 0.06^a^	10.70 ± 0.24^a^	10.56 ± 0.24^a^	0	0.58
	pHB42	R	Trp574Leu	0	6.28 ± 0.36^b^	8.66 ± 0.44^c^	7.04 ± 0.24^b^	4.31 ± 0.66^a^	

In a row, individual leaf area with different letters are significantly different at P = 0.05 significant levels. Data were means ± SE of four replicates. ^a^RCC refers to relative crowding coefficient.

All the R (carrying Pro197Leu, Pro197His, Asp376Glu or Trp574Leu) displayed greater competitiveness over S plants according the RCC values (by far less than 1.0) in terms of dry weight and leaf area. While, the pHB23 (with Pro197Ser) and S plants displayed similar competition ability in terms of dry weight (with RCC of 0.92) and leaf area (RCC of 0.96) respectively. By contrast, pHB24 plants with Pro197Thr mutation displayed stronger competitiveness than S plants in term of dry weights (with RCC of 0.62), and exhibited less competition ability than S plants in term of individual leaf area (with RCC of 1.28) (Tables 7 and 8).

## Discussion

### Effects of resistance mutation on resistance in *D. sophia*

At present, total seven resistance mutations were identified in AHAS isozymes at sites of Pro197 (Pro197Leu, His, Ser, Thr, Tyr), Asp376 (Asp376Glu) and Trp574 (Trp574Leu) in *D. sophia* populations^22–24^. In recent years, we have identified six out of seven mutations from more than sixty *D. sophia* populations. In order to minimize the differences in genetic background, individual plant with homozygous AHAS mutation (Pro197His, Pro197Ser, Pro197Thr, Pro197Leu, Asp376Glu and Trp574Leu) was purified and used in this study.

As expected, all resistance mutations caused *D. sophia* subpopulations evolve extremely high resistance to tribenuron-methyl, which were confirmed by previous experiments of dose-response, AHAS sensitivity inhibition and AHAS mutation identification^21–24^. In addition, the resistance mutations on the characterization of AHAS isozymes were conducted. The results (not include in this manuscript) also confirmed these resistance mutations played very important roles in the resistance evolution of *D. sophia* to tribenuron-methyl. Among these resistance mutations, Asp376Glu conferred the highest resistance to tribenuron-methyl. The *D. sophia* plants with Asp376Glu survived in tribenuron-methyl at dose of 37.5 g a.i. ha^−1^, which killed completely the plants carrying mutations of Pro197 and Trp574Leu (Fig. 1). Therefore, the Asp376Glu mutation is such a weak AHAS resistance mutation (in terms of growth inhibited by chlorsulfuron) in *Raphanus raphanistrum* populations comparing with the Ala122Tyr, Pro197Ser and Trp574Leu mutations^33^. Obviously, the resistance or cross-resistance patterns conferred by a given resistance mutation were depended not only on the site of mutation, but also on specific amino acid substitution. While it is not clear whether this difference is related with weed species or (and) specific herbicide. Hence, the impact of resistance-endowing mutations on resistance should be evaluated on a case-by-case basis, and generalizations should be avoided.

### Effects of resistance mutation on the classic growth of S and R plants

The RGR is the product of NAR and LAR, which depended on genetic background and environmental condition. Where, NAR is largely the net result of carbon gain (photosynthesis) and losses (respiration, exudation and volatilization) expressed per unit leaf area. The LAR is the product of a morphological component, indicating the fraction of total plant weight allocated to the leaves^34^. A high RGR help weeds occupy a large space and facilitate a rapid completion of the life cycle of a plant, which is advantageous for weeds in competitive situations. In this study, the RGR of S plants were equal or significantly higher (during 23–34 DAT and 23–39 DAT) than R plants with specific resistance mutations, which means the S plants exhibit obvious growth advantage over R plants at the early vegetative stage in the absence of stressful conditions. However, the present study was conducted only for 63 DAT and carried on under controlled conditions. There are many questions need to answer. For example, how did the resistance mutations affect plant growths in whole life history and in field conditions? Were the effects of genetic factors and environmental conditions on plant growths independent or interactional?

### Effects of resistance mutation on the competitiveness of S and R plants under condition of monoculture or admixture

The present results indicated that the different resistance mutation have different effects on the competition ability of R plants. For example, plants carrying mutation of Pro197Leu (pHB8) or Pro197His (pHB22) or Asp376Glu (pHB25) or Trp574Leu (pHB42) displayed stronger competitiveness over S plants (RCC in terms of dry weight and leaf area < 1.0). The pHB23 (Pro197Ser) exhibited similar competitiveness over S plants (RCC in terms of dry weight and leaf area ≈ 1.0) (Tables 7, 8 and Figs 3 and 4). When S and R plants mixed together, effects of S or R plants on each other were not similar. The dry weight of R plants (with Pro197Leu or Pro197Thr) reduced greatly with the increasing of R ratio from 25% to 75%. While, the dry weight of S plants was not affected except mixed with pHB23 at ratio of 25:75. The present results demonstrated the competition ability of R over S plants was affected not only by weed biotypes (S or R) and the specific resistance mutation, but also by the ratio of S or R plants in the mixtures. The dry weights of 100% R plants (carrying Pro197Leu, Pro197His, Pro197Thr, Asp376Glu or Trp574Leu) reduced greatly comparing with R plants at ratio of 75:25, 50:50 and 25:75. By contrast to R plants, the dry weight of S plants was not affected except mixed with pHB23 at ratio of 25:75. Hence, it is difficult that the R plants dominated the weed community under condition of no herbicide selection. This study was conducted in green house with single plant density, and the experiment only covered partial stages (50 DAT) of plants life cycle as other similar studies. Staged results were not necessarily in accordance with the final facts. Hence, in order to accurately estimate the resistance cost, experiments covering all life stages in a variety of biotic and abiotic environments should be conducted.

The resistance costs are considered as a basic tenet of evolutionary genetic, therefore the resistance costs are not necessary and universal in all resistance cases. The expression of resistance cost was strongly influenced by resistance mechanisms, specific resistance alleles, characteristics of target enzyme, genetic background, weed species and growth environment^2^. Hence, it is difficult to accurately assess the resistance costs in R weeds. Although a great deal of research effort has been invested to measure the resistance costs in R weed species, numerous studies were flawed. Vila-Aiub *et al*. (2009) reported only 25% of studies assessing resistance costs explicitly met the criteria of controlling genetic background^2^. To date, only a few resistance cost cases were confirmed in AHAS herbicide-resistant weed species. For example, Trp574Leu mutation of *Amaranthus powellii* can cause pleiotropic effects on the early growth and development in competitive conditions. Not only the leaves of the resistant plants were distorted and much smaller than those of S plants, one S population outperformed on R population by 7~15 times under competitive conditions^17^. In addition, the Ala205Val mutation reduced the reproductive output and fitness in resistant *S. ptychanthum* comparing with S biotype. This would likely cause S individuals to dominate in the absence of herbicide selection pressure^35^.
